# Primary umbilical endometriosis mimicking a urachal fistula: a case report

**DOI:** 10.3389/fmed.2026.1924149

**Published:** 2026-10-13

**Authors:** Xinpeng Wu, Rong Yuan, Tiange Qi, Bo Yan

**Affiliations:** 1 Department of Breast and Thyroid Surgery, Sinopharm Dongfeng General Hospital, Hubei University of Medicine, Shiyan, China; 2 Department of Operating Room, Sinopharm Dongfeng General Hospital, Hubei University of Medicine, Shiyan, China; 3 Department of Reproductive Medicine, Shenzhen Maternity and Child Healthcare Hospital, Southern Medical University, Shenzhen, China

**Keywords:** case report, cyclic umbilical bleeding, umbilical endometriosis, urachal fistula, Villar's nodule

## Abstract

**Background:**

Umbilical endometriosis is an uncommon form of extrapelvic endometriosis and may be misdiagnosed as other umbilical or urachal disorders. Cyclic umbilical bleeding or discharge is an important clinical clue, but the diagnosis is often confirmed only after surgical excision and histopathological examination.

**Case presentation:**

A 31-year-old woman presented to the hospital with a history of umbilical discharge for more than one year. The symptom occurred predominantly during menstruation and was characterized by recurrent umbilical discharge with occasional bloody secretion. Physical examination revealed mild erythema and swelling of the umbilicus, without active discharge at the time of examination. Preoperative computed tomography of the lower abdomen and pelvis showed no obvious abnormality. The initial clinical diagnosis was urachal fistula with possible umbilical infection. The patient underwent surgical excision of the umbilical lesion under general anesthesia. Intraoperatively, purulent discharge was observed at the umbilicus, and complete excision of the umbilical lesion was performed. Histopathological examination revealed endometrial glands and stroma beneath the squamous epithelium with hemosiderin deposition, while immunohistochemistry showed CD10, CK7, and PR positivity with negative/weak ER expression, supporting the diagnosis of umbilical endometriosis.

**Conclusion:**

This case emphasizes the diagnostic challenge of primary umbilical endometriosis presenting with chronic umbilical discharge. Careful assessment of menstrual history, imaging findings, and pathological examination is essential for accurate diagnosis.

## Introduction

Endometriosis is defined by the presence of endometrial-like glands and stroma outside the uterine cavity. Although the pelvis is the most common site, extrapelvic endometriosis can involve the diaphragm, thoracic cavity, abdominal wall, extrapelvic viscera, and nervous system (1). In addition, umbilical endometriosis, also known as Villar's nodule, is rare and may occur either as primary disease without previous abdominal surgery or as secondary disease associated with prior surgical procedures, it accounted for 0.5%–1.0% of all cases of endometriosis (2).

The clinical presentation of umbilical endometriosis is variable. Typical symptoms include an umbilical nodule, cyclic pain, swelling, discharge, or bleeding associated with menstruation (3). However, when the dominant symptom is umbilical discharge, the lesion may be confused with urachal anomalies, omphalitis, granuloma, epidermal inclusion cyst, or even metastatic umbilical tumors (4). This diagnostic overlap is particularly relevant in surgery practice, where patients with umbilical discharge may initially be evaluated for urachal sinus or urachal fistula.

Here, we report a case of primary umbilical endometriosis in a young woman who presented with menstruation-related umbilical discharge and bleeding and was initially diagnosed clinically as having a urachal fistula. The case emphasizes the importance of recognizing cyclic umbilical symptoms as a clue to umbilical endometriosis and highlights the diagnostic value of surgical excision followed by histopathological and immunohistochemical examination.

## Case description

A 31-year-old married woman presented with a more than one-year history of recurrent umbilical discharge, which occurred predominantly during menstruation and was occasionally accompanied by bloody secretion and mild lower back discomfort. She had received no specific treatment before admission, and the symptoms persisted.

At the time of admission, she had no fever, chills, cough, or sputum. Her appetite and sleep were generally acceptable, bowel and urinary functions were normal, and she reported no significant weight loss.

Her medical history included polycystic ovary syndrome, a history of hyperthyroidism and anxiety/depressive state. She denied a history of hypertension, coronary heart disease, diabetes mellitus, hepatitis, tuberculosis, previous surgery, trauma, or blood transfusion. She had no history of smoking or alcohol consumption. She was married and nulliparous. Her menstrual cycles were regular.

On physical examination, the patient was afebrile and hemodynamically stable. Abdominal examination showed no tenderness, rebound tenderness, or palpable abdominal mass. Urological examination revealed no obvious bilateral renal percussion pain, no tenderness along the ureteral course, and no bladder distension or suprapubic tenderness. The umbilicus showed mild erythema and swelling, with no obvious active discharge.

Preoperative lower abdominal and pelvic computed tomography did not reveal an obvious abnormality. Ultrasonography showed a hypoechoic area deep to the umbilical opening, measuring approximately 24 × 15 × 16 mm, with an unclear boundary, heterogeneous internal echogenicity, and punctate or linear blood flow signals.

Based on the clinical presentation, the initial diagnosis was urachal fistula, with possible umbilical infection and suspected endometriosis included in the differential diagnosis.

### Timeline

**Table:** 

Date	Clinical event
Approximately 1 year before admission	Menstruation-related umbilical discharge appeared, accompanied by intermittent umbilical bleeding.
2025-10-08	Lower abdominal and pelvic CT showed no obvious abnormality.
2025-10-14	Patient was admitted to the hospital. Physical examination revealed a small polypoid umbilical lesion.
2025-10-17	Surgical excision of the umbilical lesion was performed under general anesthesia.
2025-10-22	Histopathology and immunohistochemical examination confirmed umbilical endometriosis.
Follow-up（2026-01-27）	The surgical wound healed well, and no recurrence of umbilical discharge, bleeding, or menstruation-related symptoms was reported.

### Diagnostic assessment

The main diagnostic challenge in this case was distinguishing umbilical endometriosis from urachal fistula and umbilical infection. The principal diagnostic challenge was distinguishing umbilical endometriosis from a urachal anomaly and umbilical infection. Although the cyclic nature of the symptoms raised suspicion of endometriosis, the chronic discharge and subsequent finding of purulent secretion also supported an infectious or urachal etiology.

Ultrasonography demonstrated a hypoechoic lesion deep to the umbilicus with heterogeneous internal echoes and blood flow signals, supporting the presence of a localized umbilical lesion but not providing a definitive diagnosis (Figures 1A,B). The abdominal and pelvic CT scan showed no obvious sinus tract extending from the umbilical lesion into the abdominal cavity or toward the bladder (Figures 1C,D).

**Figure 1:**
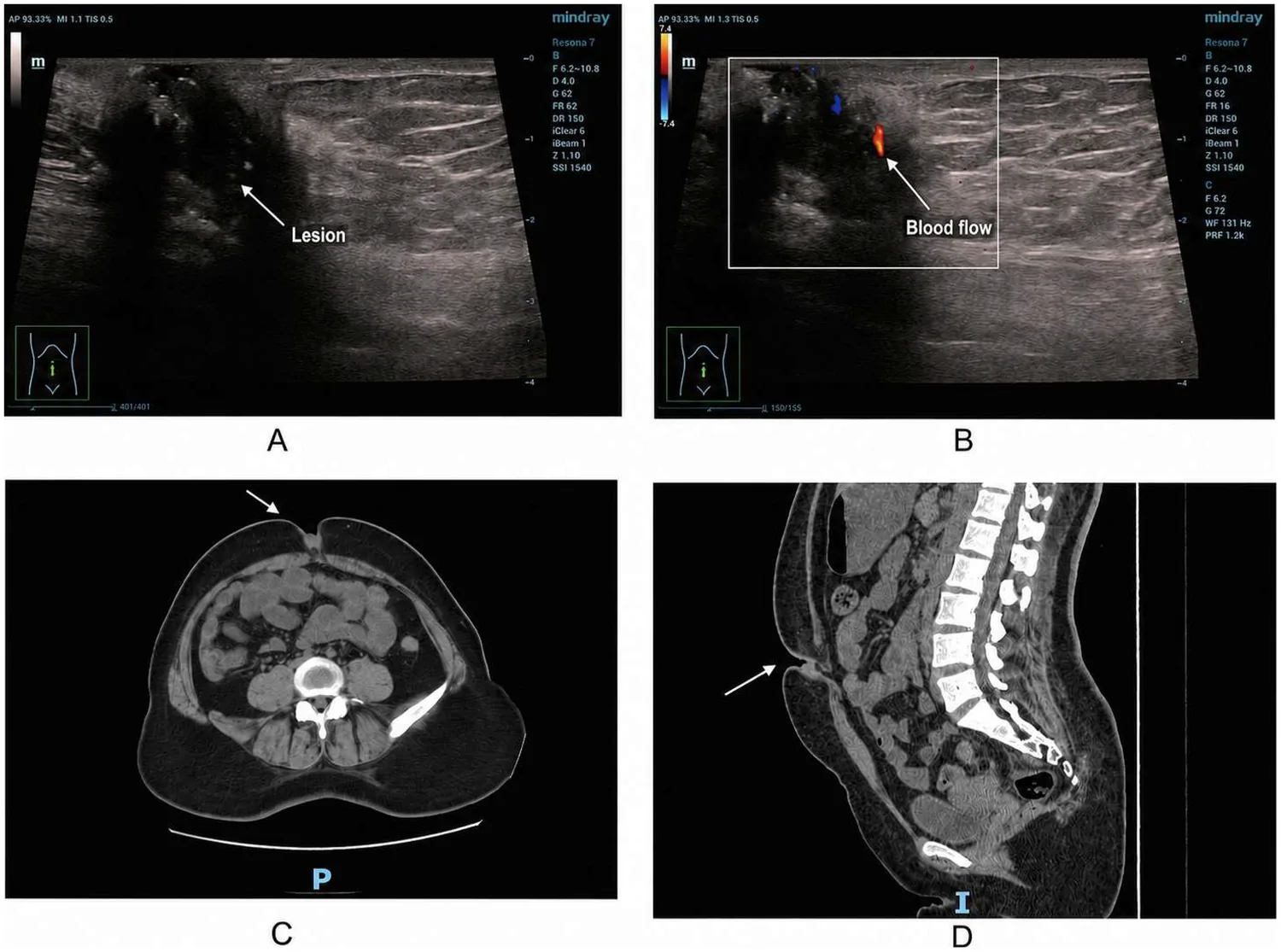
**(A)** Grayscale ultrasonography demonstrated a hypoechoic lesion deep to the umbilicus with heterogeneous internal echoes; the arrow indicates the lesion. **(B)** Color Doppler ultrasonography demonstrated internal blood flow signals within the lesion; the arrow indicates the blood flow signal. **(C,D)** Abdominal and pelvic computed tomography images. Arrows indicate the umbilical region corresponding to the clinically identified lesion. No definite sinus tract extending from the umbilicus into the abdominal cavity or toward the urinary bladder was identified.

The final diagnosis was established by histopathological and immunohistochemical examination after surgical excision. The resected surgical specimen is shown in Figure 2A. Microscopic examination demonstrated endometrial-type glands and stroma beneath the squamous epithelium, with hemosiderin deposition (Figures 2B,C). These features were consistent with umbilical endometriosis. The hemosiderin deposition also supported the history of repeated local bleeding, which corresponded to the patient's menstruation-related umbilical bleeding. Immunohistochemical staining revealed CD10-positive endometrial-type stromal cells and focal CK7-positive epithelial/glandular structures, with PR nuclear positivity in scattered cells and negative ER expression (Figures 2D–G). Taken together, the morphological and immunohistochemical findings supported the diagnosis of primary umbilical endometriosis.

**Figure 2:**
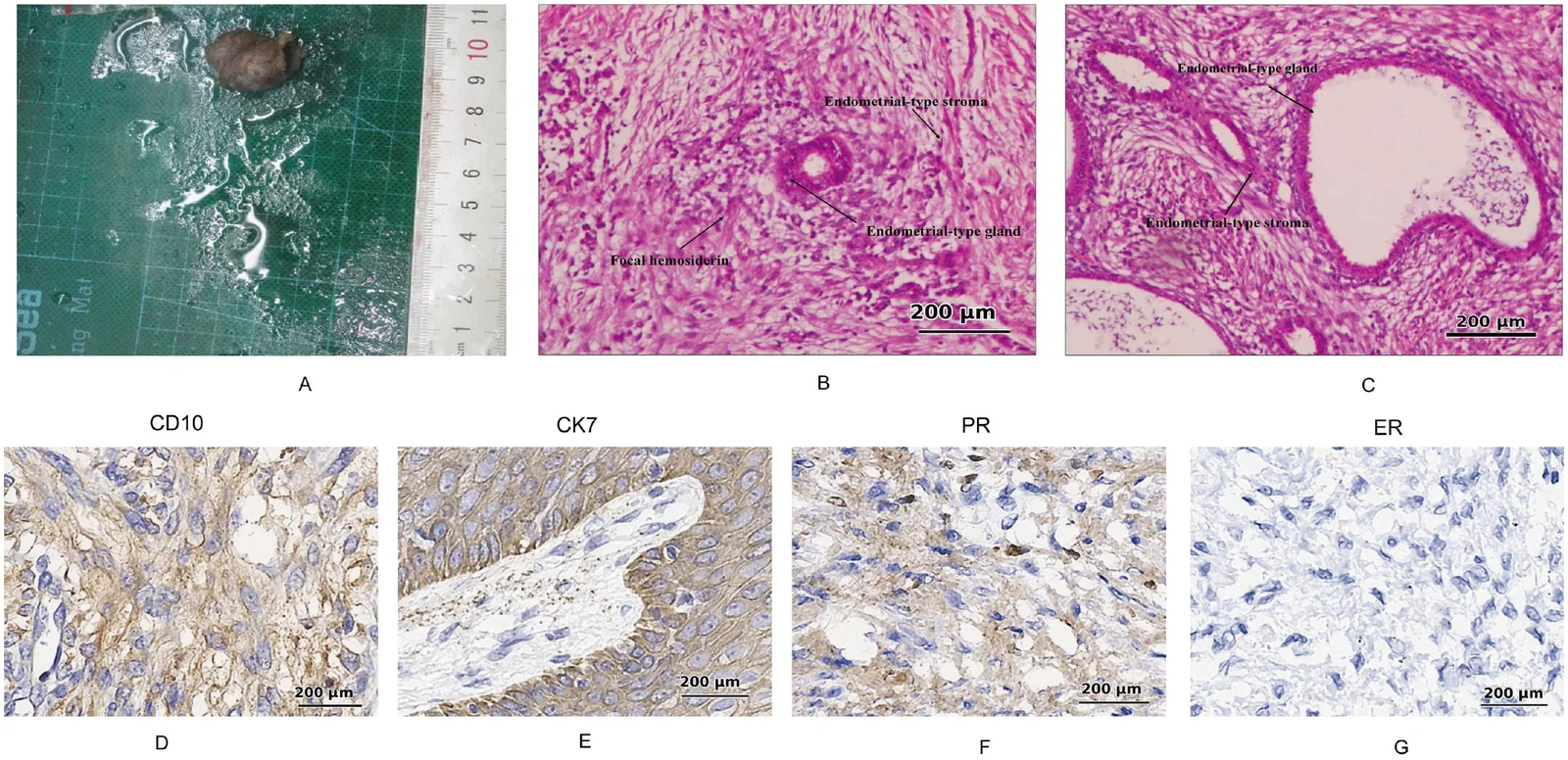
Pathological features of primary umbilical endometriosis. **(A)** Gross appearance of the excised umbilical lesion. **(B,C)** Hematoxylin and eosin staining demonstrated ectopic endometrial-type glands surrounded by endometrial-type stroma, with hemosiderin deposition and hemorrhagic changes. Representative arrows indicate endometrial-type glands, endometrial-type stroma, and hemosiderin deposition. **(D–G)** Immunohistochemical staining showed CD10-positive endometrial stromal cells, CK7-positive glandular epithelial structures, PR nuclear positivity, and negative/weak ER expression. These morphological and immunohistochemical findings supported the diagnosis of primary umbilical endometriosis. Scale bars: 200 μm.

### Therapeutic intervention

The patient underwent umbilical lesion excision under general anesthesia. After routine disinfection and draping, a circumumbilical incision of approximately 0.5 cm was made. The skin and subcutaneous tissues were dissected layer by layer. Purulent discharge was noted at the umbilicus. The lesion was carefully dissected to the base of the umbilicus and completely excised. The incision was irrigated repeatedly, and the wound was closed layer by layer with absorbable sutures. The intraoperative blood loss was approximately 2 mL, and the operation was completed uneventfully. No change in the therapeutic strategy was required because the lesion was completely excised and the patient had an uneventful postoperative recovery.

Postoperatively, the patient received oxygen therapy, electrocardiographic monitoring, intravenous antibiotics, fluid therapy, and symptomatic treatment. Her postoperative recovery was uneventful, and she was discharged after clinical improvement.

### Follow-up and outcomes

The postoperative wound healed well, and no recurrence of umbilical discharge, bleeding, or menstruation-related symptoms was observed during the 3 month follow-up period.

## Discussion

The main clinical lesson from this case is that menstruation-related umbilical discharge should raise suspicion of umbilical endometriosis in reproductive-age women. Although cyclic discharge or bleeding is a characteristic clue, the diagnosis can be challenging when the predominant manifestation resembles other umbilical disorders (5).

In the present case, the diagnosis was particularly challenging because the clinical picture overlapped with that of a urachal anomaly and umbilical infection. The intraoperative finding of purulent secretion further reinforced the initial suspicion of an infectious or urachal process. Nevertheless, the cyclic nature of the symptoms remained an important clue, and the definitive diagnosis was ultimately established by histopathological examination.

Chronic umbilical discharge has a broad differential diagnosis, including urachal anomalies, infection, benign umbilical lesions, and metastatic tumors (6–8). In reproductive-age women, the association between symptoms and menstruation should raise suspicion of umbilical endometriosis (3).

Previous reports of primary umbilical endometriosis have most commonly described women of reproductive age presenting with an umbilical nodule, cyclic pain, swelling, or bleeding associated with menstruation (9, 10). Similar to these reports, our patient was of reproductive age, had no history of abdominal surgery, and exhibited menstruation-related umbilical symptoms, supporting the diagnosis of primary rather than secondary umbilical endometriosis. However, the present case differed in that chronic umbilical discharge, rather than a painful or visibly bleeding umbilical nodule, was the predominant manifestation. In addition, purulent secretion was observed intraoperatively, which further suggested an infectious or urachal etiology and contributed to the initial diagnosis of urachal fistula with possible umbilical infection. This atypical presentation may therefore broaden the recognized clinical spectrum of primary umbilical endometriosis and illustrates why the condition may be overlooked in patients presenting primarily with umbilical discharge.

Imaging findings in umbilical endometriosis are variable and are not always diagnostic (11). In our patient, ultrasonography demonstrated a localized heterogeneous hypoechoic lesion with internal vascular signals, whereas CT failed to demonstrate a definite urachal tract or other obvious abnormality. This discrepancy between ultrasonography and CT is also clinically relevant, as the absence of a characteristic CT abnormality does not exclude superficial umbilical endometriosis. Therefore, imaging should be interpreted together with the cyclic nature of symptoms, particularly when no definite communication with the bladder or intra-abdominal urachal remnant is identified.

Histopathologically, umbilical endometriosis is characterized by ectopic endometrial glands and stroma, with hemosiderin deposition providing supportive evidence of repeated hemorrhage (12). In this case, the pathological findings corresponded with the history of cyclic umbilical bleeding. Immunohistochemistry further supported the diagnosis by demonstrating CD10-positive stromal cells, CK7-positive glandular structures, and PR positivity. Although ER expression was negative or weak, this finding does not exclude the diagnosis and should be interpreted together with clinical and morphological findings.

Surgical excision is the preferred treatment for umbilical endometriosis because it removes the lesion, provides tissue for definitive diagnosis, and reduces the risk of recurrence. Complete excision is particularly important when the lesion is symptomatic, infected, or difficult to distinguish from other umbilical or urachal diseases.

This case report has several limitations. First, it describes a single patient, and therefore the findings cannot be generalized to all patients with umbilical endometriosis. Second, the follow-up period was limited, so the long-term risk of recurrence could not be fully assessed. Third, although ultrasonography and computed tomography were performed, preoperative imaging did not establish a definitive diagnosis, and additional imaging such as magnetic resonance imaging was not performed. Fourth, although umbilical infection was included in the differential diagnosis and purulent discharge was observed intraoperatively, microbiological culture and antimicrobial susceptibility testing of the discharge were not performed; therefore, concomitant bacterial infection could not be definitively confirmed or excluded. Finally, the immunohistochemical findings should be interpreted together with the characteristic histological morphology and clinical cyclic symptoms, particularly because ER expression was negative or weak in this case. In addition, dedicated intraoperative photographs were not available because the definitive diagnosis of umbilical endometriosis was established only after postoperative histopathological examination.

In conclusion, this case demonstrates that umbilical endometriosis can mimic urachal fistula or umbilical infection. For reproductive-age women presenting with umbilical discharge or bleeding, especially when symptoms are related to menstruation, umbilical endometriosis should be considered. Surgical excision, histopathological and immunohistochemical examination remain essential for definitive diagnosis and treatment.

## Patient perspective

The patient reported that the recurrent umbilical discharge and bleeding, especially during menstruation, caused anxiety and affected her daily life. After surgical treatment and explanation of the final pathological diagnosis, she expressed relief that the cause of the symptoms had been clarified. She agreed that sharing her clinical experience may help other patients with similar symptoms receive an earlier diagnosis.

## Data Availability

The original contributions presented in the study are included in the article/Supplementary Material, further inquiries can be directed to the corresponding authors.
